# The relationship between retinal vascular tortuosity and retinal vasculitis

**DOI:** 10.1186/s12348-025-00512-7

**Published:** 2025-10-01

**Authors:** Xiaoyan Zhang, Frances Andrea Anover, Jia-Horung Hung, Ngoc Trong Tuong Than, Azadeh Mobasserian, Aim-On Saengsirinavin, Negin Yavari, Dalia El Feky, Anh Ngoc Tram Tran, Osama Elaraby, Jingli Guo, Irmak Karaca, Woong-Sun Yoo, Amir Akhavanrezayat, Chi Mong Christopher Or, Diana V. Do, Quan Dong Nguyen

**Affiliations:** 1 Spencer Center for Vision Research, Byers Eye Institute, Stanford University School of Medicine, Palo Alto, USA; 2 Eye Hospital, China Academy of Chinese Medical Sciences, Beijing, China

**Keywords:** Arterial tortuosity, Venous tortuosity, Arterial and venous tortuosity, And retinal vasculitis

## Abstract

**Aims:**

This study investigates the association between retinal vascular tortuosity and retinal vasculitis.

**Methods:**

A retrospective review of medical records was conducted for 135 patients diagnosed with retinal vasculitis at our institution from June 2022 to June 2024. The presence and type of retinal vascular tortuosity were assessed, and logistic regression analysis was used to evaluate associations with viral infections, autoimmune conditions, and other clinical features.

**Results:**

Of 256 patients with posterior uveitis, 135 patients were identified with retinal vasculitis, and 37 (27.4%) exhibited retinal vascular tortuosity. Specifically, 24 patients presented with arterial tortuosity, 5 with venous tortuosity, and 8 with both arterial and venous tortuosity. Logistic regression analysis revealed that arterial tortuosity was significantly associated with posterior synechiae, while venous tortuosity was primarily observed in patients with birdshot chorioretinopathy. Combined arterial and venous tortuosity was more commonly observed in patients with viral infections, toxoplasmosis, or psoriasis. Notably, after inflammation was controlled, retinal vascular tortuosity improved. However, the average recovery times varied between arterial tortuosity, venous tortuosity, and the combination of both.

**Conclusion:**

Retinal vascular tortuosity is prevalent in retinal vasculitis and is associated with specific infectious etiologies and clinical features. It may serve as a prognostic marker for disease severity and treatment planning.

## Introduction

Retinal vasculitis is a condition characterized by inflammation of the retinal blood vessels. It can occur as an isolated ocular finding, or in association with other signs of concurrent intraocular inflammation. It is often associated with various conditions, including immune-mediated disorders, infections, or malignancies [1]. It is routinely diagnosed based on the characteristic clinical findings of vascular sheathing, cotton wool spots, as well as intraretinal hemorrhages. Retinal vasculitis causes vascular leakage visible on fluorescein angiography (FA) and can result in retinal edema, exudation, and cystoid macular edema, all of which can contribute to vision loss [1]. 

The normal vessels of the retinal vascular tree are straight or gently curved. One distinctive feature of retinal vasculitis is vascular tortuosity, which refers to the abnormal twisting and curvature of blood vessels. Vascular tortuosity can serve as an indicator of underlying pathology and may also reflect disease severity and treatment response.

Factors such as inflammation, hypoxia, shear stress, mechanical pressure fluctuations, and vascular changes (e.g., endothelial and smooth muscle cell proliferation, degeneration of the elastic layer) contribute to the development and progression of tortuosity in arteries, veins, capillaries, or a combination of these vessel types [2]. 

In recent years, there has been growing interest in understanding the relationship between retinal vascular tortuosity and various systemic diseases, including infections and autoimmune disorders [3, 4]. Inflammatory processes in retinal vasculitis may affect vascular structures, leading to tortuosity. Elevated levels of vascular endothelial growth factor and other cytokines (e.g., interleukin-6 and interleukin-8) lead to the disruption of the endothelial zonula occludens, endothelial fragmentation, formation of fenestrations, and degeneration of the basement membrane. Other factors, such as hyperviscosity and hypercoagulable states, can alter the path of retinal vessels and cause vessel occlusions, given that these are associated with endothelial damage, stasis, and disruptions in blood flow [2]. 

Understanding the associations between retinal vascular tortuosity and retinal vasculitis is crucial for improving disease management and guiding clinical decision-making.

This study aims to explore the relationship between retinal vascular tortuosity and retinal vasculitis, particularly focusing on the role of infections, specific clinical manifestations, and its potential as a prognostic marker.

## Methods

This was a retrospective review of medical records from patients diagnosed with retinal vasculitis at our institution between June 2022 and June 2024. A total of 135 patients were included in the analysis. All patients included in the study were consecutive cases. No patients were excluded due to inadequate image quality. Data were collected from medical records, including demographics, ocular and systemic history, clinical features, imaging results, and laboratory findings.

Retinal vascular tortuosity was assessed using fundus photography and FA. Patients were classified based on the type of vascular tortuosity: arterial, venous, or both.

Clinical features evaluated included: band keratopathy, posterior synechiae, anterior uveitis, vitritis, optic inflammation, white dot syndrome, vascular occlusion, hemorrhage, macular edema.

Follow-up intervals were adjusted based on the severity of inflammation and tortuosity. Patients with active inflammation and tortuosity were asked to return every two months, while those with controlled inflammation and tortuosity returned approximately every six months. The assessment of tortuosity was performed consistently at these follow-up visits.

### Statistical analysis

Logistic regression analysis was performed using IBM SPSS Version 30.0 to assess the relationship between retinal vascular tortuosity and various clinical factors, including viral infections, autoimmune conditions, and other systemic diseases.

## Results

A total of 135 patients with retinal vasculitis were included in the study, and the etiological factors associated with retinal vasculitis are presented in Table 1. Among these 135 patients, 37 (27.4%) exhibited retinal vascular tortuosity. Of these 37 patients, 27 had bilateral involvement. In cases of bilateral tortuosity, the eye with the more severe presentation was selected for analysis. The average age of patients in the tortuosity group was 36.5 ± 21.1 years (range: 8–82), compared to 40.3 ± 22.1 years (range: 8–87) in the non-tortuosity group. Of the 37 patients with tortuosity, 14 (37.8%) were male; of the 98 patients without tortuosity, 38 (38.8%) were male.

**Fig. 1:**
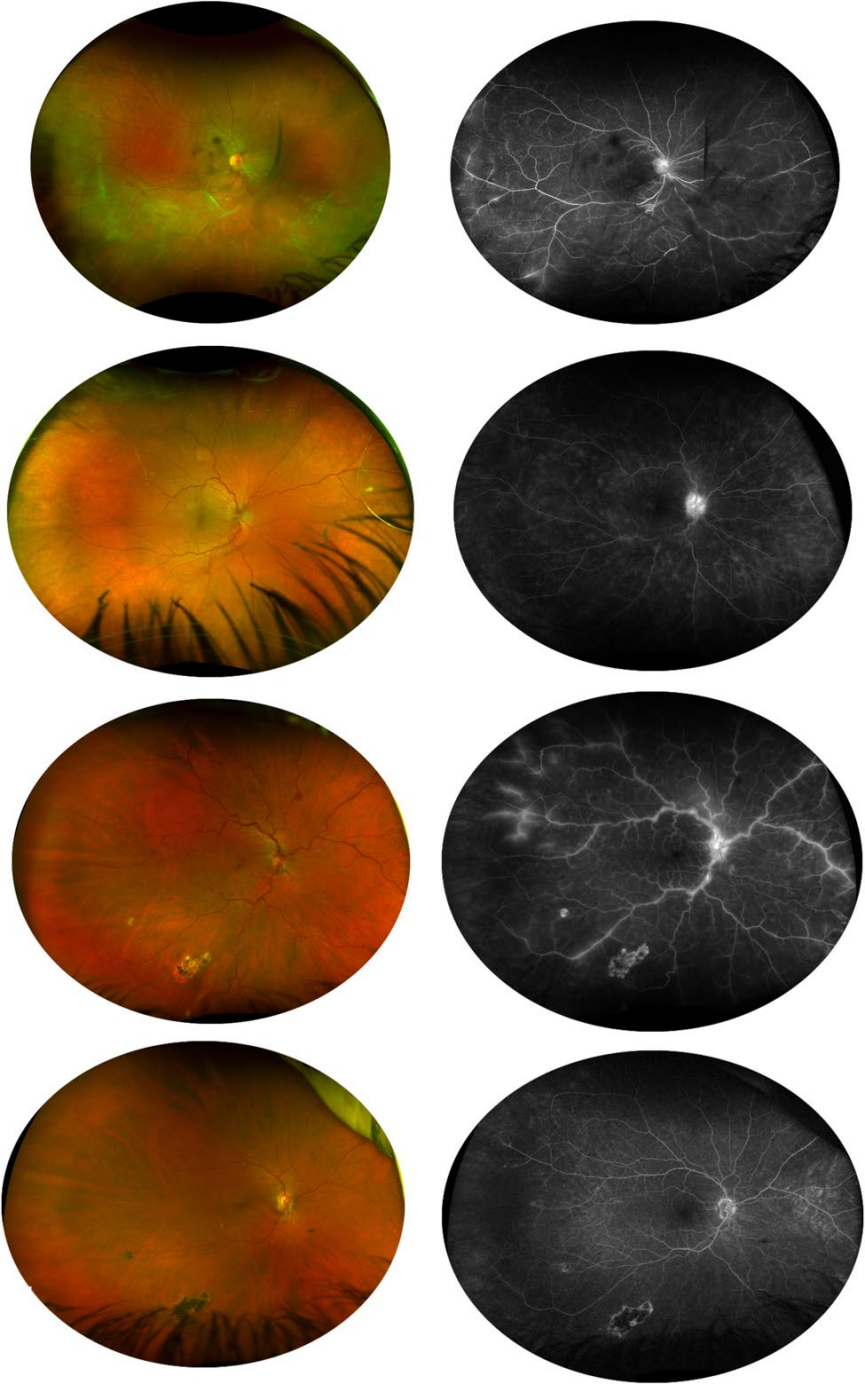
Fundus photography and FA illustrating arterial tortuosity (**A**, **B**), venous tortuosity (**C**, **D**), and both arterial and venous tortuosity (**E**, **F**). The changes of both arterial and venous tortuosity after inflammation control (**G**, **H**)

The distribution of tortuosity types was as follows: 24 patients with arterial tortuosity, 5 patients with venous tortuosity, and 8 patients with both arterial and venous tortuosity (Fig. 1). Notably, after inflammation was controlled, venous tortuosity improved, whereas arterial tortuosity remained unchanged (Fig. 1).

**Table 1:** Etiologies of Retinal Vasculitis

Etiologies of retinal vasculitis	Number of patients
Birdshot chorioretinopathy	3
Viral infections	7
Toxoplasmosis	2
Psoriasis	2
Sarcoidosis	3
Tuberculosis	13
Syphilis	6
Juvenile Idiopathic Arthritis	7
Acanthamoeba Keratitis	3
Systemic Lupus Erythematosus	3
Behçet’s Disease	1

Logistic regression analysis revealed several key findings: Arterial tortuosity was strongly associated with the presence of posterior synechiae (odds ratio [OR]: 2.77, 95% confidence interval [CI] = 1.02–7.46) (Table 2). Venous tortuosity was predominantly observed in patients with birdshot chorioretinopathy (OR: 16, 95% CI = 1.19–215.11) (Table 3). Both arterial and venous tortuosity were more commonly observed in patients with viral infections (OR: 8.93, 95% CI = 1.80–44.22), toxoplasmosis (OR: 18, 95% CI = 1.02–318.87), or psoriasis (OR: 18, 95% CI = 1.02–318.87) (Table 4).

**Table 2:** Risk factors for arterial tortuosity in retinal vasculitis

Risk factor	OR	95% CI
Posterior synechiae	2.77	1.02–7.46

**Table 3:** Risk factors for venous tortuosity in retinal vasculitis

Risk factor	OR	95% CI
Birdshot chorioretinopathy	16	1.19–215.11

**Table 4:** Risk factors for both arterial and venous tortuosity in retinal vasculitis

Risk factors	OR	95% CI
Viral infections	8.93	1.80–44.22
Toxoplasma	18.00	1.02–318.87
Psoriasis	18.00	1.02–318.87

Logistic regression analysis showed no significant association between tortuosity and gender (OR: 1.04; 95% CI: 0.48–2.27), age (OR: 0.99; 95% CI: 0.97–1.01), Best-Corrected Visual Acuity (OR: 2.39, 95% CI: 0.76–7.50), or hypertension (OR: 1.06; 95% CI: 0.20–5.73).

The mean time to recovery of arterial tortuosity was 5.3 ± 8.9 months (range: 2–40 months), and for venous tortuosity, it was 16 ± 15.0 months (range: 2–38 months). In patients with both arterial and venous tortuosity, venous changes resolved, while arterial tortuosity persisted beyond 24 months. In this subgroup, the mean time to venous recovery was 7.3 ± 7.1 months (range: 2–20 months).

## Discussion

Our study highlights the complex relationship between retinal vascular tortuosity and retinal vasculitis. The presence of vascular tortuosity in patients with retinal vasculitis suggests significant vascular remodeling in response to inflammation. It has been suggested that the severity of many retinal diseases, and the progression of retinopathies, could be inferred from the degree of tortuosity of the blood vessel network [5]. 

Arterial tortuosity was found to be significantly associated with posterior synechiae, suggesting that tortuosity may reflect the severity of inflammation within the eye. In severe cases of vascular inflammation, arterial blood flow is disrupted, leading to increased pressure and permeability of the vessels. This may cause the vessel walls to become distorted, contributing to the characteristic twisting and curving seen in arterial tortuosity [6]. 

The association between venous tortuosity and birdshot chorioretinopathy aligns with prior studies showing that venous changes are common in this condition [7]. Birdshot chorioretinopathy, which is associated with the HLA-A29 genetic marker, suggests a genetic predisposition to retinal vasculitis and vascular changes. The persistence of venous tortuosity, despite the control of inflammation, suggests that some vascular changes may be irreversible or may take longer to resolve, warranting further research into the underlying pathophysiological mechanisms.

Combined arterial and venous tortuosity was more common in patients with viral infections, toxoplasmosis, and psoriasis, all of which are associated with chronic inflammatory processes. A previous study found there was venous tortuosity, and ischemic retinal whitening in ocular toxoplasmosis [8]. Viral infections (e.g., cytomegalovirus, herpesvirus) can lead to persistent inflammation in the retina, resulting in vascular abnormalities [9]. Similarly, Toxoplasma gondii can cause severe retinal inflammation (toxoplasmic retinitis), resulting in vascular remodeling and tortuosity. Psoriasis, as a systemic inflammatory disease, is known to affect vascular health, including the retina. Notably, after inflammation was controlled, venous tortuosity improved, likely due to the normalization of blood flow and reduced vessel wall stress.

These findings underscore the importance of considering underlying infectious and inflammatory causes in patients with retinal vasculitis exhibiting vascular tortuosity. Clinicians should monitor for these conditions, particularly when both arterial and venous tortuosity are present. This is especially relevant in tortuosity associated with occlusive vasculitis, which commonly leads to lasting structural complications such as epiretinal membrane formation and cystoid macular edema. Additionally, late-stage tortuosity changes may be associated with vascular occlusion and remodeling processes, such as telangiectasias, microaneurysms, and ischemia-induced neovascularization. These changes can result in complications such as recurrent vitreous hemorrhage, tractional retinal detachment, iris neovascularization, and neovascular glaucoma [1]. 

Our observations suggest that vascular remodeling may follow different temporal dynamics depending on the vessel type. The prolonged persistence of arterial tortuosity could be attributed to structural differences in vascular wall or variations in hemodynamic stress responses between arteries and veins. Further longitudinal studies with larger cohorts are warranted to better characterize these patterns and to understand the underlying mechanisms driving differential recovery timelines.

A limitation of the study is its small sample size. Further research is warranted to explore the detailed changes in vascular tortuosity during follow-up after treatment.

## Conclusion

Retinal vascular tortuosity is a prevalent feature in patients with retinal vasculitis and is associated with specific infectious etiologies and clinical features. Identifying vascular tortuosity may serve as a prognostic marker to assess disease severity, guide treatment decisions, and improve patient outcomes. Future studies should focus on longitudinal assessments to better understand the implications of vascular tortuosity in retinal vasculitis.

## Data Availability

No datasets were generated or analysed during the current study.
